# Prognostic Impact of Positive Peritoneal Lavage Cytology on Resectable Pancreatic Body and Tail Cancer: A Retrospective Study

**DOI:** 10.1007/s00268-022-06818-0

**Published:** 2022-11-10

**Authors:** Taro Mashiko, Toshihito Ogasawara, Yoshihito Masuoka, Shigenori Ei, Shinichiro Takahashi, Kenichi Hirabayashi, Masaki Mori, Kazuo Koyanagi, Seiichiro Yamamoto, Toshio Nakagohri

**Affiliations:** 1 Department of Gastroenterological Surgery, Tokai University School of Medicine, 143 Shimokasuya, Isehara, Kanagawa 259-1193 Japan; 2 Department of Diagnostic Pathology, Faculty of Medicine, University of Toyama, 2630, Sugitani, Toyama, 930-0194 Japan

## Abstract

**Background:**

The prognostic impact of positive peritoneal lavage cytology on pancreatic cancer is unclear. Therefore, this study aimed to evaluate its impact in resectable pancreatic body and tail cancer.

**Methods:**

Between January 2006 and December 2019, 97 patients with pancreatic body and tail cancer underwent peritoneal lavage cytology and curative resection at our institution. We analyzed the impact of positive peritoneal lavage cytology on clinicopathological factors and on the prognosis of pancreatic body and tail cancer.

**Results:**

Malignant cells were detected in 14 patients (14.4%) using peritoneal lavage cytology. In these patients, the tumor diameter was significantly larger (*p* < 0.001) and anterior serosal invasion (*p* = 0.034), splenic artery invasion (*p* = 0.013), lympho-vessel invasion (*p* = 0.025), and perineural invasion (*p* = 0.008) were significantly more frequent. The R1 resection rate was also significantly higher in patients with positive peritoneal lavage cytology than in negative patients (*p* = 0.015). Positive peritoneal lavage cytology had a significantly poor impact on overall survival (*p* = 0.001) and recurrence-free survival (*p* < 0.001). This cytology was also an independent poor prognostic factor for recurrence (*p* = 0.022) and was associated with peritoneal dissemination and liver metastasis.

**Conclusions:**

Positive peritoneal lavage cytology is considered to be indicative of more systemic disease in patients with resectable pancreatic body and tail cancer than in patients with negative peritoneal lavage cytology. Early detection of pancreatic cancer before it develops micrometastases is important to improve prognosis, and CY+ patients require more intensive multimodality treatment than standard treatment for resectable pancreatic cancer.

## Introduction

Pancreatic cancer is currently the 4th leading cause of cancer-related death in the USA. With the number of patients increasing, it is expected to become the second leading cause by 2030 [1, 2]. Despite the development of multidisciplinary treatment for pancreatic cancer, the high rate of recurrence leads to a 5-year survival rate < 20% [3–5].

Peritoneal lavage cytology is widely used for diagnosing and staging gastric, ovarian, and endometrial cancers [6–8]. However, the prognostic impact of positive lavage cytology (CY +) on pancreatic cancer is controversial, with some reports indicating that it is a poor prognostic factor [9–11] and others indicating that it has no effect [12–14]. Positive peritoneal lavage cytology is reportedly more common in pancreatic body and tail than in pancreatic head cancer [15]. However, few studies have focused on its prognostic impact in this population because fewer numbers of such patients undergo resection compared to those with pancreatic head cancer [16]. Therefore, this study aimed to evaluate the prognostic impact of positive peritoneal lavage cytology on pancreatic body and tail cancer.

## Materials and methods

### Patients

We retrospectively reviewed 123 consecutive patients who underwent radical resection for preoperatively resectable pancreatic body and tail cancer between January 2006 and December 2019 at Tokai University Hospital, Japan. Staging was performed based on the Union for International Cancer Control (UICC) tumor-node-metastasis (TNM) classification, 8^th^ edition [17]. Based on this guideline, R0 margins were defined as no tumor cells at any of the resection margins, and R1 as tumor cells being present at the margin without macroscopic residual tumor.

The diagnosis of resectability of pancreatic cancer was based on preoperative imaging findings in accordance with the National Comprehensive Cancer Networks (NCCN) guidelines [18]. Postoperative complications were evaluated using the Clavien–Dindo classification [19].

### Neoadjuvant treatment and surgery

For pancreatic body and tail cancer, distal pancreatectomy with regional lymph node dissection was performed. Laparoscopic surgery has been increasingly performed for pancreatic cancer and has been adopted at our department. However, during the study period, essentially all pancreatic cancers were resected by laparotomy. There were no major changes in the surgical technique over the study period. Neoadjuvant chemotherapy for resectable pancreatic cancer has been increasingly adopted. During the study period, the decision on neoadjuvant treatment was made by the attending physician, and upfront surgery was generally performed. Radiotherapy is not administered for resectable pancreatic cancer at our department.

### Peritoneal lavage cytology

Peritoneal washing and cytological analysis were performed after laparotomy. Normal saline (100 mL) was introduced into the abdominal cavity and gently agitated; the washing solution was collected from the pouch of Douglas. Smears were prepared from the centrifuged deposit and examined by two experienced pathologists after Papanicolaou and Giemsa staining. If one cancer cell was present, positive peritoneal lavage cytology was diagnosed.

### Postoperative follow-up and adjuvant chemotherapy

All patients underwent routine postoperative surveillance. Patients undergoing postoperative adjuvant chemotherapy also underwent tumor marker measurement [carcinoembryonic antigen (CEA) and colorectal carcinoma antigen (CA19-9)] monthly for the first 6 months after surgery, then every 3 months until 3 years after surgery, and every 6 months thereafter. Patients not undergoing postoperative adjuvant chemotherapy underwent tumor marker measurement every 3 months. Chest and abdominal computed tomography was performed every 3 months after surgery for the first 3 years, every 6 months for the following 2 years, and annually thereafter. The choice of postoperative adjuvant chemotherapy depended on the attending physician; before 2016, gemcitabine-based chemotherapy was introduced following the result of the CONKO-001 trial [20], and since the results of the JASPAC01 trial, S-1 therapy has been the mainstay [21].

## Statistical analysis

All statistical analyses were performed using SPSS (version 26.0; Chicago, IL). The Chi-square and Mann–Whitney U tests were used to analyze categorical and continuous variables, respectively. Overall survival (OS) and recurrence-free survival (RFS) were analyzed using the Kaplan–Meier method, and statistical significance was evaluated using the log-rank test. Univariate and multivariate Cox proportional hazard regression analyses were conducted to identify the prognostic factors for pancreatic body and tail cancer. Multivariate analyses were performed for variables showing *p* <0.05 in the univariate analyses. A *p*-value < 0.05 was considered to be statistically significant. The cut-off values for preoperative CEA and CA19-9 that could predict prognosis were calculated using receiver operating characteristic curves.

## Results

**Fig. 1:**
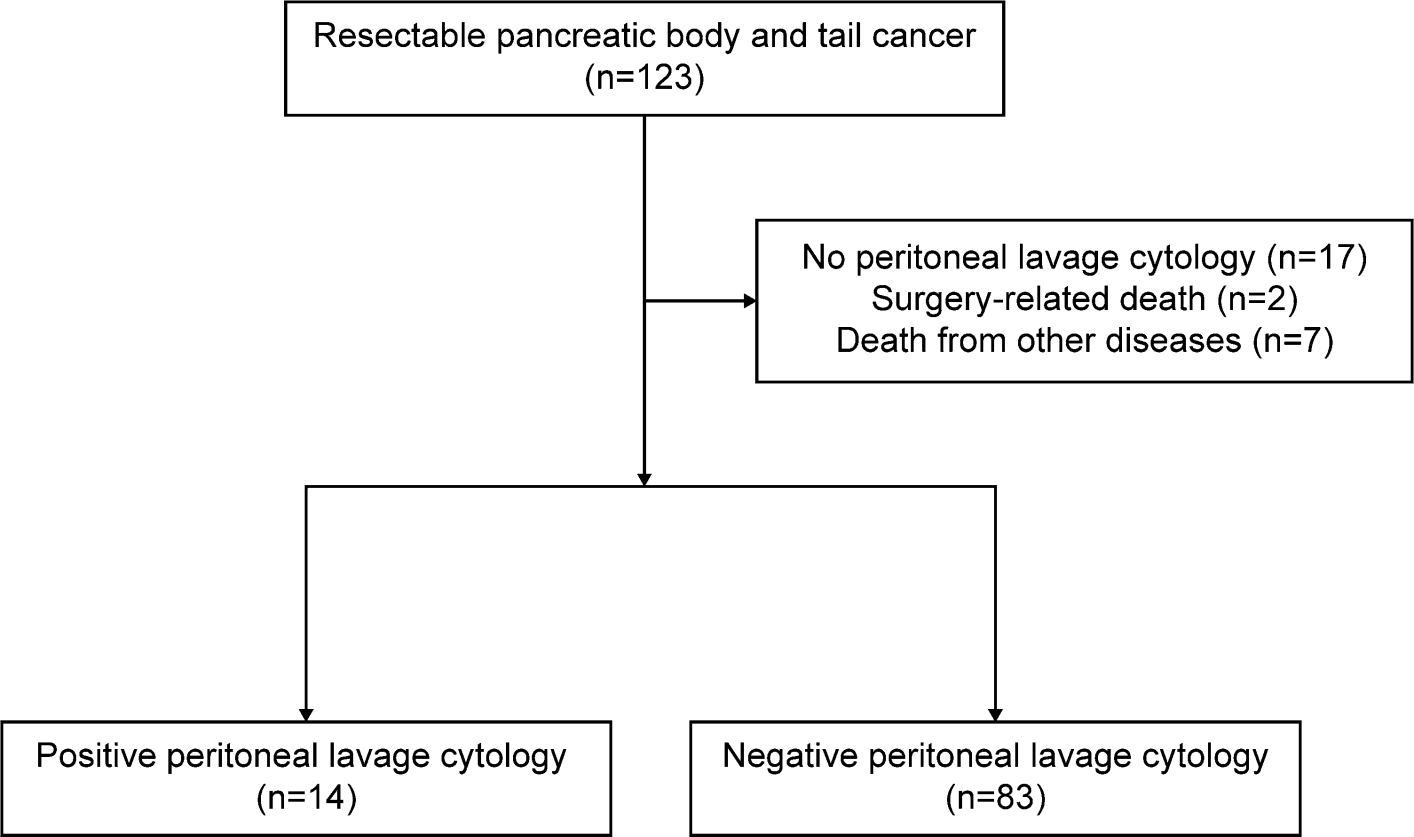
Flowchart of patients with resected pancreatic body and tail cancer

After excluding 17 cases wherein peritoneal lavage cytology was not performed, two surgery-related deaths, and seven deaths due to other diseases, 97 cases were included in the final analysis (Fig. 1). Of the 97 patients, 51 were males and 46 were females, with a median age of 69 years (range: 45–84 years). The median observation period was 36 months (range: 5–104 months). The TNM stage 1A, 1B, 2A, 2B, and 3 were present in 21, 24, 8, 30, and 14 patients, respectively. R0 resection was achieved in 80 cases (82.5%). For adjuvant chemotherapy, patients received S-1 (*n* = 51, 52.6%), gemcitabine (*n* = 17, 17.5%), and gemcitabine combined with S-1 (*n* = 10, 10.3%). Adjuvant chemotherapy was completed in 61 cases (69.3%). There were 14 CY + (14.4%) and 83 CY− patients (85.6%). The patients’ baseline characteristics are shown in Table 1.

**Table 1:** Baseline characteristics of patients with resected pancreatic body and tail cancer

Variables		
Age, years, median (range)		69 (45–84)
Sex (male:female)		51:46:00
Hemoglobin (g/dl), median (range)		13.1 (8.8–16.4)
Albumin (g/dl), median (range)		4.1 (1.6–4.7)
CEA (ng/mL), median (range)		4.1 (0.8–52.0)
CA19-9 (U/mL), median (range)		60.9 (6.5–2768.1)
Neoadjuvant chemotherapy, *n* (%)		
Gemcitabine+S-1		3 (3.1)
Operation time (min), median (range)		221 (93–500)
Blood loss (ml), median (range)		556 (74–3482)
Blood transfusion (Yes), *n* (%)		12 (12.4)
Portal vein resection, *n* (%)		6 (6.2)
Combined resection of other organs, *n* (%)		31 (32.0)
Postoperative pancreatic fistula, *n* (%)		50 (51.5)
Complication (Clavien–Dindo classification), *n* (%)	1,2	45 (46.4)
	> 3	52 (53.6)
Tumor size (mm), median (range)		30 (11–90)
Anterior serosal invasion, *n* (%)		59 (60.8)
Retroperitoneal invasion, *n* (%)		82 (84.5)
Extrapancreatic nerve plexus invasion, *n* (%)		34 (35.1)
Portal vein/Splenic vein invasion, *n* (%)		37 (38.1)
Splenic artery invasion, *n* (%)		12 (12.4)
Lympho-vessel invasion (positive), *n* (%)		76 (78.4)
Vascular invasion (positive), *n* (%)		26 (26.8)
Perineural invasion (positive), *n* (%)		44 (45.4)
Lymph node metastasis (positive), *n* (%)		44 (45.4)
Peritoneal lavage cytology (positive), *n* (%)		14 (14.4)
Pathologic T category, *n* (%)		
	T1	24 (24.7)
	T2	49 (50.5)
	T3	24 (24.7)
Pathologic N category, *n* (%)		
	N0	53 (54.6)
	N1	31 (32.0)
	N2	13 (13.4)
Resected margin status, *n* (%)		
	R0	80 (82.5)
	R1	17 (17.5)
Tumor differentiation, *n* (%)		
	Well	44 (45.4)
	Moderate	43 (44.3)
	Poor	4 (4.1)
	Adenosquamous	4 (4.1)
	Mucinous	2 (2.1)
Pathologic UICC-stage, *n* (%)		
	1A	21 (21.7)
	1B	24 (24.7)
	2A	8 (8.3)
	2B	30 (30.9)
	3	14 (14.4)
Adjuvant chemotherapy, *n* (%)		
	S-1	51 (52.6)
	Gemcitabine	17 (17.5)
	Gemcitabine+S-1	10 (10.3)
Completion of adjuvant chemotherapy, *n* (%)		61 (62.9)

*UICC*: Union for international cancer control, *CEA*: carcinoembryonic antigen, CA19-9: colorectal cancer antigen

A comparison of clinicopathological factors in the CY + and CY− groups is shown in Table 2. Preoperative CA19-9 level was significantly higher in the CY + group (*p* = 0.027). There was a higher rate of blood transfusions in the CY + group (*p* = 0.013), but no significant differences in operative time, blood loss, complications, or length of hospital stay. Among histopathological factors, tumor diameter was significantly larger in the CY + group (*p* < 0.001), and anterior serosal invasion (*p* = 0.034), splenic artery invasion (*p* = 0.013), lympho-vessel invasion (*p* = 0.025), and perineural invasion (*p* = 0.008) were significantly more common. The R1 resection rate was significantly higher in the CY + group (*p* = 0.015).

**Table 2:** Comparison of clinicopathological findings between the CY+ and CY− groups

Variables	CY+(*n* = 14)	CY− (*n* = 83)	*p*-value
Age, years, median (range)	68 (45–84)	70 (45–84)	0.622
Sex (Male/Female)	07-Jul	44/39	0.53
Hemoglobin (g/dl), median (range)	12.5 (10.0–14.7)	13.1 (8.8–15.8)	0.177
Albumin (g/dl), median (range)	4.0 (3.3–4.6)	4.1 (1.6–4.7)	0.47
CEA (ng/mL), median (range)	5.9 (1.2–52.0)	4.0 (0.8–43.8)	0.35
CA19-9 (U/mL), median (range)	222.1 (15.9–1736.4)	52.7 (0.5–2768.1)	0.027
Operation time (min), median (range)	240.5 (102–400)	210 (74–3071)	0.107
Portal vein resection, *n* (%)	2 (14.3)	4 (4.8)	0.207
Blood loss (ml), median (range)	737.5 (81–3482)	543 (93–500)	0.23
Blood transfusion (Yes), *n* (%)	5 (35.7)	7 (8.4)	0.013
Complication (Clavien–Dindo classification > 3a)	8 (57.1)	44 (53.0)	0.503
Postoperative pancreatic fistula (Grade > B)	8 (57.1)	42 (50.6)	0.436
Duration of hospitalization (day), median (range)	26 (19–39)	24 (11–92)	0.456
Tumor size (mm), median (range)	52.5 (23–70)	28 (11–90)	< 0.001
Anterior serosal invasion (positive), *n* (%)	12 (85.7)	47 (51.8)	0.034
Retroperitoneal invasion (positive), *n* (%)	14 (100)	68 (81.9)	0.079
Extrapancreatic nerve plexus invasion (positive), *n* (%)	7 (50.0)	27 (32.5)	0.167
Splenic/Portal vein invasion (positive), *n* (%)	7 (50.0)	30 (36.1)	0.243
Splenic artery invasion (positive), *n* (%)	5 (35.7)	7 (8.4)	0.013
Lympho-vessel invasion (positive), *n* (%)	14 (100)	62 (74.7)	0.025
Vascular invasion (positive), *n* (%)	5 (35.7)	21 (25.3)	0.304
Perineural invasion (positive), *n* (%)	11 (78.6)	33 (39.8)	0.008
Lymph node metastasis (positive), *n* (%)	8 (57.1)	36 (43.4)	0.252
UICC-T category (T1,2/T3)	04-Oct	69/14	< 0.001
UICC-stage (1,2/3)	12-Feb	72/11	0.596
Tumor differentiation (Well, Moderately/Poorly, Other)	13-Jan	73/10	0.505
Resected margin status (R0/R1)	08-Jun	72/11	0.015
Neoadjuvant chemotherapy (Yes), *n* (%)	0	3 (3.6)	0.623
Adjuvant chemotherapy (Yes), *n* (%)	10 (71.4)	67 (80.7)	0.317
Completion of adjuvant chemotherapy, *n* (%)	7 (50.0)	55 (66.3)	0.191

**Fig. 2:**
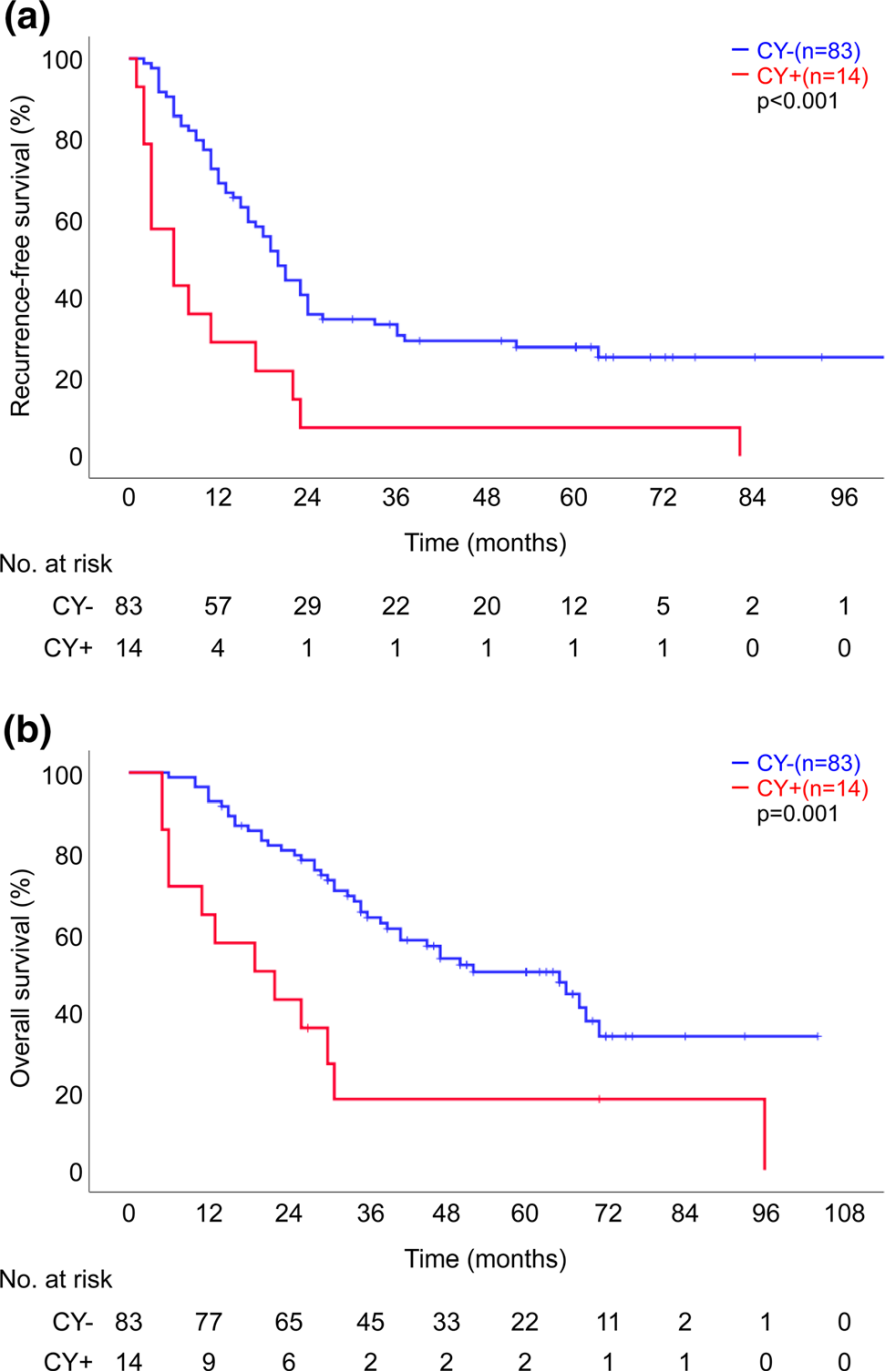
**a** Comparison of recurrence-free survival (RFS) between patients with positive lavage cytology (CY+ and negative lavage cytology (CY−) **b** Comparison of overall survival (OS) between patients with positive lavage cytology (CY+) and negative lavage cytology (CY−)

The 1−, 3−, and 5-year survival rates were 64.3%, 17.9%, and 17.9% in the CY + group and 92.8%, 63.5%, and 49.8% in the CY− group, respectively. The median OS of the CY + and CY− groups was 19.0 [95% confidence interval (CI): 2.5–35.5] and 52.0 (95% CI: 31.9–72.1) months, respectively (*p* = 0.001). The 1−, 3−, and 5-year RFS rates were 28.6%, 7.1%, and 7.1% in the CY + group and 68.7%, 30.3%, and 27.4% in the CY− group, respectively. The median RFS of the CY + and CY− groups was 6.0 (95% CI: 0.6–11.4) and 20.0 (95% CI: 17.1–22.9) months, respectively (*p* < 0.001). Both OS and RFS were worse in the CY + group (Fig. 2).

When comparing recurrence between the groups, relapse was noted in all the patients in the CY + group, showing a significantly higher rate than that in the CY− group (*p* = 0.016). In one case in the CY + group, a tumor developed in the residual pancreas after 5 years, and biopsy showed adenocarcinoma. The initial surgery achieved R0 margins. No genetic testing was performed, and whether this was a residual recurrence or an iatrogenic pancreatic cancer was unclear. However, its occurrence does not seem to be directly related to the fact that peritoneal lavage cytology was positive. Liver metastasis and peritoneal dissemination tended to be more common in the CY + group (Table 3). First-line chemotherapy after relapse is shown in Table 4. Gemcitabine plus nab-paclitaxel was administered more frequently in the CY− group (*p* = 0.007). Five patients (35.7%) in the CY + group and nine (15.0%) in the CY− group received palliative care due to advanced age or poor general condition after relapse (*p* = 0.085).

**Table 3:** Recurrence sites in the CY+ and CY− groups

Variables	CY +	CY−	*p*-value
	(*n* = 14)	(*n* = 83)	
Recurrence	14 (100)	60 (72.3)	0.016
Local	7 (50)	27 (32.5)	0.167
Liver	6 (42.9)	13 (15.7)	0.028
Peritoneum	6 (42.9)	10 (12.0)	0.011
Lung	1 (7.1)	11 (13.3)	0.453
Distant lymph node	0	5 (6.0)	0.451

**Table 4:** Chemotherapy after recurrence in the CY + and CY− groups

Regimen	CY +	CY−	*p*-value
	(*n* = 14)	(*n* = 60)	
Gemcitabine	2 (14.3)	9 (15.0)	0.656
S-1	3 (21.4)	5 (8.3)	0.169
Gemcitabine plus S-1	2 (14.3)	9 (15.0)	0.656
Gemcitabine plus nab-paclitaxel	1 (14.3)	27 (45.0)	0.007
FOLFIRINOX	1 (7.1)	0	0.189

In the univariate analysis, the following factors were significantly associated with poor OS: preoperative CEA level (> 4.0 ng/ml), preoperative CA19-9 level (> 65.2 U/ml), positive extrapancreatic plexus invasion, positive venous invasion, positive perineural invasion, CY + , and failure to complete postoperative adjuvant chemotherapy. Multivariate analysis showed that the CA19-9 level (> 65.2 U/ml) [hazard ratio (HR): 2.58, 95% CI: 1.46–4.57, *p* = 0.001)], positive perineural invasion (HR: 3.38, 95% CI: 1.90–6.02, *p* < 0.001), UICC-T3, that is tumor diameter > 40 mm (HR: 2.02, 95% CI: 1.09–3.58, *p* = 0.025), and failure to complete adjuvant chemotherapy (HR: 2.75, 95% CI: 1.54–4.93, *p* = 0.001) were independent poor prognostic factors. On the other hand, CY + was not significantly associated with OS (Table 5).

**Table 5:** Prognostic factors of overall survival in pancreatic body and tail cancer by univariate and multivariate analyses

Factors	Univariate analysis		Multivariate analysis	
	HR (95% CI)	*p*-value	HR (95% CI)	*p*-value
Age (> 69)	0.79 (0.46–1.36)	0.393		
Sex (Female)	1.02 (0.60–1.74)	0.934		
Albumin (< 4.1 g/dl)	1.34 (0.74–2.40)	0.332		
CEA (> 4.0 ng/ml)	1.95 (1.13–3.37)	0.016	1.37 (0.73–2.57)	0.328
CA19-9 (> 65.2 U/ml)	2.57 (1.49–4.44)	0.001	2.58 (1.46–4.57)	0.001
Operation time (> 212 min)	1.36 (0.79–2.34)	0.272		
Blood loss (> 556 ml)	1.69 (0.98–2.92)	0.058		
Blood transfusion (Yes)	1.21 (0.54–2.70)	0.647		
Portal vein resection (Yes)	1.06 (0.33–3.42)	0.927		
Combined resection of other organs (Yes)	1.58 (0.90–2.77)	0.112		
Clavien–Dindo classification (> 3a)	1.32 (0.77–2.26)	0.31		
Anterior serosal invasion (positive)	1.48 (0.58–2.58)	0.171		
Retroperitoneal invasion (positive)	2.70 (0.98–7.49)	0.056		
Extrapancreatic nerve plexus invasion (positive)	2.00 (1.16–3.43)	0.013	1.11 (0.59–2.10)	0.746
Portal/Splenic vein invasion (positive)	1.60 (0.94–2.74)	0.085		
Splenic artery invasion (positive)	1.93 (0.94–3.95)	0.074		
Lympho-vessel invasion (positive)	1.79 (0.84–3.78)	0.131		
Vascular invasion (positive)	2.04 (1.17–3.56)	0.012	0.92 (0.48–1.76)	0.746
Perineural invasion (positive)	3.13 (1.80–5.45)	< 0.001	3.38 (1.90–6.02)	< 0.001
Peritoneal lavage cytology (CY) (positive)	2.91 (1.51–5.63)	0.001	1.70 (0.82–3.53)	0.154
Lymph node metastasis (positive)	1.20 (0.70–2.04)	0.516		
UICC-T3	2.43 (1.37–4.29)	0.002	1.98 (1.09–3.58)	0.025
UICC-Stage3	1.23 (0.60–2.53)	0.566		
Resection margin status (R1)	1.36 (0.68–2.71)	0.381		
Tumor differentiation (Poor/others)	0.72 (0.26–1.99)	0.523		
Neoadjuvant chemotherapy (No)	0.76 (0.19–3.14)	0.708		
Adjuvant chemotherapy (No)	1.58 (0.83–3.01)	0.163		
Completion of adjuvant chemotherapy (No)	2.29 (0.26–1.99)	0.003	2.75 (1.54–4.93)	0.001

Univariate analysis for RFS showed that CEA level (> 4.0 ng/ml), CA19-9 level (> 65.2 U/ml), combined resection of other organs, positive anterior serosal invasion, positive retroperitoneal invasion, positive extrapancreatic plexus invasion, positive splenic artery invasion, positive perineural invasion, CY + status, positive lymph node metastasis, UICC-T3, UICC-stage 3, R1 resection, and failure to complete adjuvant chemotherapy were poor prognostic factors. Multivariate analysis showed that CA19-9 level (> 65.2 U/ml) (HR: 3.91, 95% CI: 2.30–6.66, *p* <  0.001), positive perineural invasion (HR: 2.43, 95% CI: 1.47–4.02, *p* = 0.001), UICC-T3 (HR:1.98, 95% CI: 1.03–3.69, *p* = 0.035), CY + (HR: 2.09, 95% CI: 1.11–3.93, *p* = 0.022), R1 resection (HR: 2.92, 95% CI: 1.58–5.42, *p* = 0.001), and failure to complete adjuvant chemotherapy (HR: 3.18, 95% CI: 1.90–5.32, *p* <  0.001) were poor prognostic factors (Table 6). In terms of RFS, CY + was found to be an independent poor prognostic factor in resectable pancreatic body and tail cancer.

**Table 6:** Prognostic factors of recurrence-free survival in pancreatic body and tail cancer by univariate and multivariate analyses

Factors	Univariate analysis		Multivariate analysis	
	HR (95%CI)	*p*-value	HR (95% CI)	*p*-value
Age (> 69)	0.78 (0.46–1.36)	0.393		
Sex (Female)	0.77 (0.49–1.22)	0.274		
Albumin (< 4.1 g/dl)	1.10 (0.68–1.78)	0.706		
CEA (> 4.0 ng/ml)	2.30 (1.43–3.70)	0.001	1.11 (0.59–2.11)	0.743
CA19-9 (> 65.2 U/ml)	3.28 (2.04–5.28)	< 0.001	3.91 (2.30–6.66)	< 0.001
Operation time (> 212 min)	1.25 (0.79–1.97)	0.351		
Blood loss (> 556 ml)	1.54 (0.97–2.44)	0.068		
Blood transfusion (Yes)	0.93 (0.45–1.95)	0.856		
Portal vein resection (Yes)	0.90 (0.33–2.45)	0.825		
Combined resection of other organs (Yes)	2.00 (1.24–3.21)	0.004	1.48 (0.88–2.48)	0.137
Clavien–Dindo classification (> 3a)	0.90 (0.56–1.41)	0.622		
Anterior serosal invasion (positive)	1.91 (1.17–3.12)	0.01	1.16 (0.67–2.03)	0.6
Retroperitoneal invasion (positive)	3.85 (1.55–9.56)	0.004	2.52 (0.95–6.63)	0.062
Extrapancreatic nerve plexus invasion (positive)	2.11 (1.32–3.38)	0.002	0.95 (0.51–1.78)	0.869
Portal/Splenic vein invasion (positive)	1.51 (0.95–2.39)	0.082		
Splenic artery invasion (positive)	2.15 (1.13–4.12)	0.02	0.85 (0.41–1.78)	0.851
Lympho-vessel invasion (positive)	1.68 (0.92–3.05)	0.092		
Vascular invasion (positive)	1.63 (0.98–2.70)	0.058		
Perineural invasion (positive)	2.16 (1.37–3.43)	0.001	2.43 (1.47–4.02)	0.001
Peritoneal lavage cytology (CY) (positive)	2.86 (1.58–5.15)	< 0.001	2.09 (1.11–3.93)	0.022
Lymph node metastasis (positive)	1.91 (1.17–3.12)	0.006	1.06 (0.60–1.87)	0.851
UICC-T3	2.39 (1.43–3.98)	0.001	1.98 (1.03–3.69)	0.035
UICC-Stage3	2.17 (1.17–4.02)	0.014	1.30 (0.67–2.49)	0.448
Resection margin status (R1)	2.29 (1.31–4.01)	0.004	2.92 (1.58–5.42)	0.001
Tumor differentiation (Poor/others)	1.10 (0.55–2.21)	0.788		
Neoadjuvant chemotherapy (No)	1.10 (0.27–4.46)	0.902		
Adjuvant chemotherapy (No)	1.55 (0.89–2.70)	0.121		
Completion of adjuvant chemotherapy (No)	1.90 (1.19–3.03)	0.008	3.18 (1.90–5.32)	< 0.001

*HR*: Hazard ratio, *CI*: Confidence interval *UICC*: Union for international cancer control, *CEA*: Carcinoembryonic antigen, CA19-9: Colorectal cancer antigen

## Discussion

In this study, CY + in pancreatic body and tail cancer was significantly associated with poor RFS but not OS. Peritoneal dissemination and liver metastases were significantly more common in the CY + group.

Intraoperative peritoneal lavage cytology is used in the diagnosis of various carcinomas.

CY + has been reported to be a poor prognostic factor in gastric, colorectal and cervical cancers [22–24]. However, its prognostic impact depends on the specific cancer, with no effect observed in ovarian and biliary tract cancers [25, 26]. In recent years, CY+ in pancreatic cancer has been considered to have the same status as stage 4 metastatic disease according to the NCCN, UICC and American Joint Committee on Cancer (AJCC) guidelines [17, 18, 27]. Conversely, CY findings are not included in the staging system of the Japan Pancreas Society based on previous studies, and pancreatic cancers are not considered to be unresectable [28].

Pancreatic body and tail cancer has a higher rate of metastasis at the time of diagnosis and poorer prognosis than pancreatic head cancer [29, 30]. While pancreatic head cancer is usually detected subsequent to symptoms such as obstructive jaundice and cholangitis, patients with pancreatic body and tail cancer are less likely to present with symptoms and often have more locally advanced disease at the time of diagnosis. This is one reason why peritoneal washing cytology is often positive in pancreatic body and tail cancer. In the present study, the CY+ group had significantly larger tumors; and more common anterior tissue invasion, lympho-vessel and perineural invasion, and R1 resections, indicating a greater systemic disease burden.

Although the accuracy of diagnostic imaging to determine locally advanced pancreatic cancer has been improving, occult distant metastases that are not detected preoperatively are still observed intraoperatively [31, 32]. In recent years, staging laparoscopy has been performed to determine peritoneal lavage cytology, detect distant metastases, and avoid non-curative surgery [33, 34]. Traverso et al. reported that, among patients diagnosed with unresectable locally advanced pancreatic cancer on preoperative computed tomography, staging laparoscopy found distant metastases in 28% of pancreatic head and 53% of pancreatic body and tail cancers [15]. Moreover, Krabicak et al. reported that staging laparoscopy showed unexpected distant metastases in 46% of pancreatic head and 66% of pancreatic body and tail cancers (*p*= 0.011). They further reported that pancreatic body and tail cancer diameter ≥ 42 mm is an independent risk factor for peritoneal dissemination, similar to our findings in the CY+ group. They concluded that staging laparoscopy should be performed in patients with large pancreatic body and tail cancer because of the high risk of peritoneal dissemination [35].

The size of pancreatic cancers has been reported to be an important factor in recurrence and prognosis. Although the prognosis worsens with increasing tumor size, smaller tumor still exhibits frequent recurrences and metastases. Ansari et al. reported that distant metastasis occurred in 30.6% of patients with tumors ≤0.5 cm [36]. According to Marchegiani et al., tumors > 20 mm in diameter have a significantly poorer prognosis, with more positive lymph node metastases, poorly differentiated tissue, perineural invasion, and R1 resections, and should be treated with neoadjuvant chemotherapy. They also found that preoperative imaging should be evaluated carefully as it underestimates actual tumor size by nearly 20% [37]. Haeno et al. developed a computational model that predicts metastasis at the time of diagnosis using factors such as primary tumor size. They found that pancreatic cancer growth is initially exponential. This suggests that tumor size at diagnosis is important for predicting metastasis and prognosis. They further stated that it is more important to initiate treatment to control cell proliferation than to perform surgery [38]. Tumor diameter > 40 mm was also independently associated with OS and RFS in this study.

Gemcitabine plus nab-paclitaxel and FOLFIRINOX [a combination of drugs, including: FOL – folinic acid, F – fluorouracil, Irin – irinotecan, and Ox – oxaliplatin] has become the standard treatment for unresectable or recurrent pancreatic cancer. The median OS for these treatments is reported to be 8.5 and 11.1 months, respectively [39, 40]. The median OS in resectable CY+ patients reportedly ranges from 8.0 to 23.8 months [9, 10, 14, 41], a better prognosis than that of patients with pancreatic cancer with distant metastases. On the other hand, locally advanced pancreatic cancer treated with chemotherapy or chemoradiotherapy alone with gemcitabine plus nab-paclitaxel or FOLFIRINOX has shown improved outcomes, with survival ranging from 10.0 to 32.7 months. This prognosis is better than that for CY+ resected cases [42, 43]. Neoadjuvant chemotherapy is being increasingly used in resectable pancreatic cancer [44]. In order to improve the prognosis of CY+ patients, it is necessary to reconsider treatment methods, such as using more potent gemcitabine plus nab-paclitaxel or FOLFIRINOX postoperatively, in addition to neoadjuvant treatment. However, early detection, before the development of micrometastases, remains the most important issue. Recently, liquid biopsy involving tumor cells, circulating tumor DNA, microRNA; and artificial intelligence have been used for early detection of pancreatic cancer [45–47].

This study has some limitations. First, this was a retrospective single-center study with a relatively small number of patients. Second, the detection of cancer cells may be underestimated because peritoneal lavage cytology was only performed in the pouch of Douglas. The detection rate is reportedly higher when peritoneal lavage cytology is performed at multiple locations [48]. In addition, many reports use 100–200 ml of saline solution for pancreatic cancer [11–13, 16], compared to 10–1000 ml or more for other cancers [49, 50], which may also affect the detection rate. In addition, reverse-transcription polymerase chain reaction reportedly increases detection sensitivity [51]. Finally, the validation in postoperative adjuvant chemotherapy and treatment after recurrence may have led to selection bias. Therefore, a multi-institutional prospective study is needed to clarify the clinical impact and best treatment of CY+ patients with pancreatic body and tail cancer.

In conclusion, positive peritoneal lavage cytology in patients with resectable pancreatic body and tail cancer is considered to be indicative of greater systemic disease. Detection of pancreatic cancer before this occurs remains crucial. Standard treatment for resectable pancreatic cancer does not provide satisfactory prognosis, and these patients require more optimal and intensive multimodality treatment.
